# Supplementary material for: A1CF Binding to the p65 Interaction Site on NKRF Decreased IFN-β Expression and p65 Phosphorylation (Ser536) in Renal Carcinoma Cells
Source: Int J Mol Sci. 2024 Mar 22;25(7):3576. doi: 10.3390/ijms25073576 (PMC11011687; doi:10.3390/ijms25073576)
Supplement: Supplementary file 1 [file ijms-25-03576-s001.zip › ijms-2894599-supplementary.pdf]

International Journal of
Molecular Sciences

Supplementary Materials:

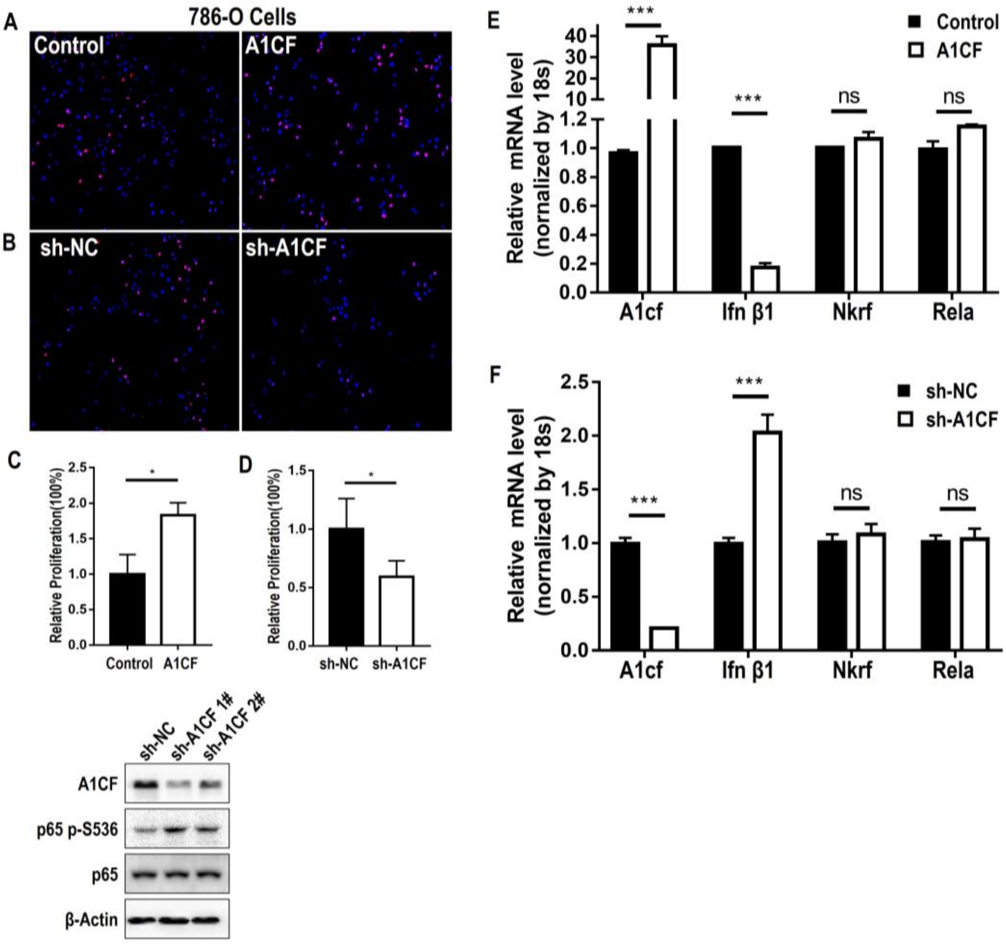

Figure S1. A1CF controlled cell growth through negative regulation of IFN-β via NF-κB signaling activation A-D Growth of 786-O stable cells were measured by EdU assay. Results were expressed as mean ± SEM from 3 replicate well. p-values were calculated by Student t-test, *p < 0.05. (**A**). Merged image of EdU staining (red) and Hoechst staining (blue) for 786-O A1CF overexpression stable cells. (B). Merged image of EdU staining (red) and Hoechst staining (blue) for 786-O A1CF deficiency stable cells. (**C, D**). Statistical analysis of 786-O cell proliferation. Values were presented as mean ± SEM (n = 3). p-values were calculated by Student t-test, * p < 0.05. (**E**). 786-O cells expressing sh-NC, sh-A1CF1#, sh-A1CF2# were examined by western blotting with the indicated antibodies. (**F, G**). mRNA levels of indicated genes in 786-O stable cells were measured by RT-qPCR. The data are presented as means ± SD from triplicate samples. ***p < 0.001.

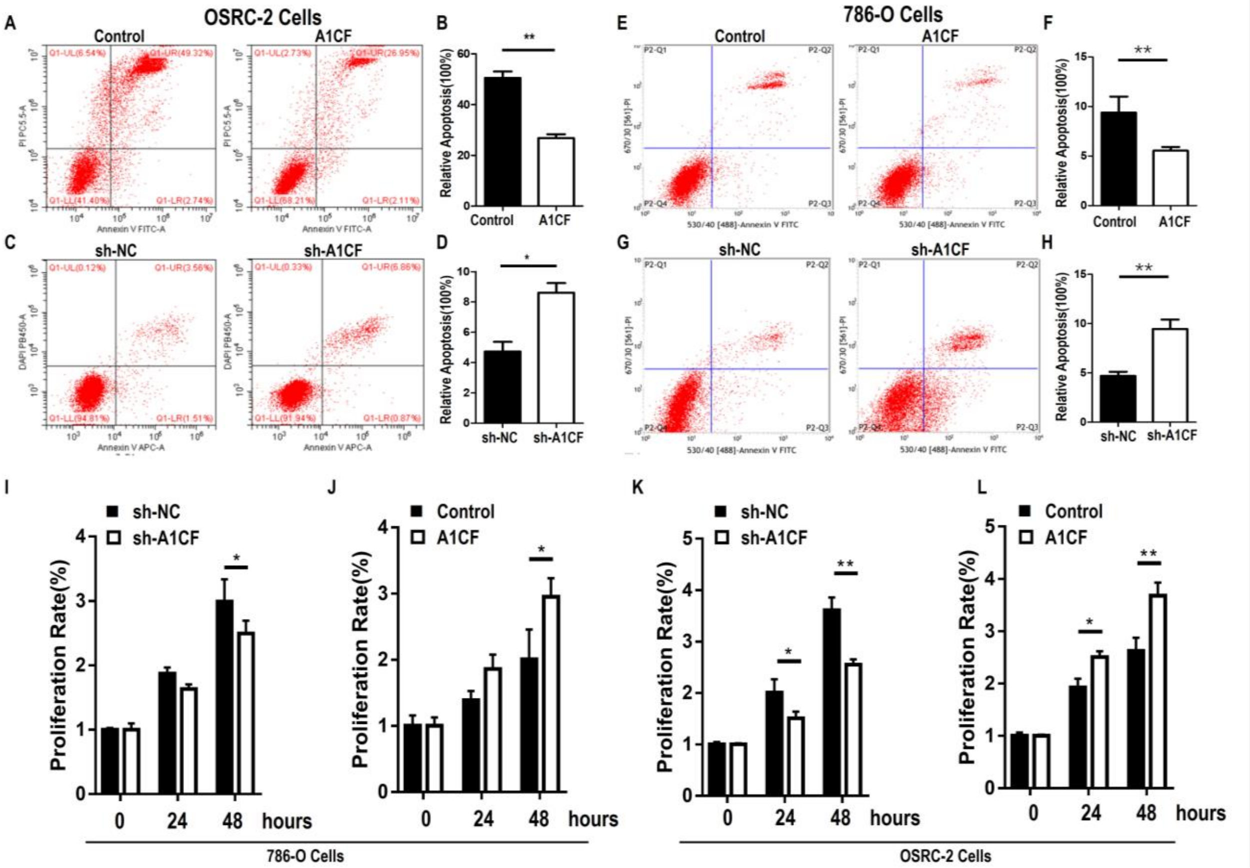

Figure S2. A1CF promoted proliferation and inhibited apoptosis in stable renal cells. **(A-H)** Apoptosis of stable renal cancer cells were measured by flow cytometry with Annexin V-PI staining. Results were expressed as mean ± SEM from 3 replicates. p-values were calculated by Student t-test, $*p < 0.05$, $**p<0.01$. (**A**). Flow cytometry analysis was performed to evaluate the percentage of apoptotic cells in OSRC-2 A1CF overexpression stable cells. (**C**). Flow cytometry analysis was performed to evaluate the percentage of apoptotic cells in OSRC-2 A1CF deficiency stable cells. (**B, D**). Statistical analysis of cell apoptosis in OSRC-2 stable cell lines and histogram was drawn in GraphPad Prism 5. Values were presented as mean ± SEM (n = 3). p-values were calculated by Student t-test, $* p < 0.05$**.** (**E**). Flow cytometry analysis was performed to evaluate the percentage of apoptotic cells in 786-O A1CF overexpression stable cells. (**G**). Flow cytometry analysis was performed to evaluate the percentage of apoptotic cells in 786-O A1CF knockdown stable cells.

(**F, H**). Statistical analysis of cell apoptosis in 786-O stable cell lines and histogram was drawn in GraphPad Prism 5. Values were presented as mean ± SEM (n = 3). p-values were calculated by Student t-test, $** p < 0.01$. (**I-L**). Proliferation of stable renal cancer cells were measured by MTT assay. Results were expressed as mean ± SEM from 6 replicate well. p-values were calculated by Student t-test, $* p < 0.05$; $**p < 0.01$. (**I, J**). 786-O stable cell lines indicated above were seeded on 96-well plates and cell viability was measured by MTT assays at 0, 24, 48 hours. (**K, L**). OSRC-2 stable cell lines indicated above were seeded on 96-well plates and cell viability was measured by MTT assays at 0, 24, 48 hours.

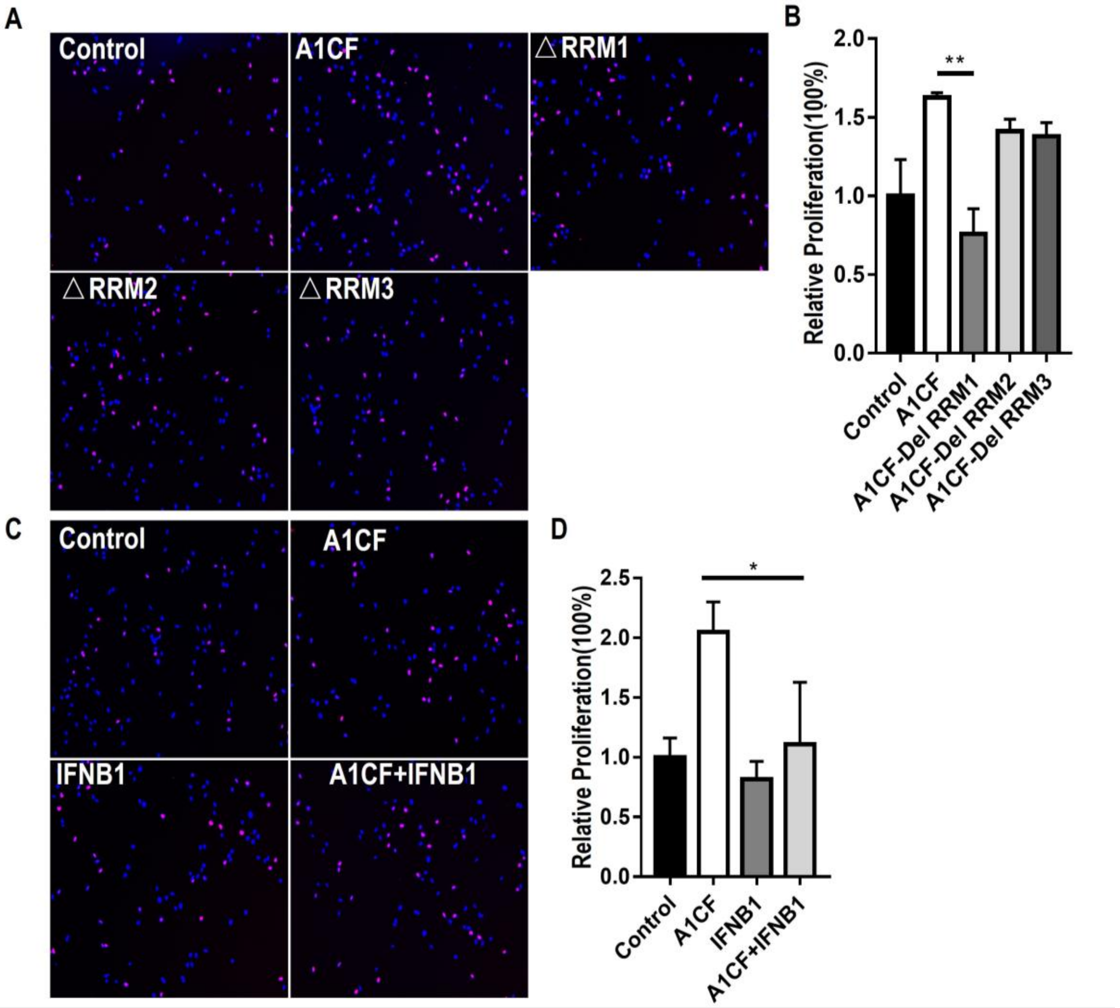

Figure S3. Disrupting the A1CF-NKRF interaction changed the renal cancer cell proliferation measured by EdU assay through A1CF-NKRF- NF-κB- IFN-β axis. (**A-D**). Growth of 786-O cells expressing the indicated plasmids were measured by EdU assay. Results were expressed as mean ± SEM from 3 replicate well. p-values were calculated by Student t-test, *$p < 0.05$. (**A**). Merged image of EdU staining (red) and Hoechst staining (blue) for 786-O cells expressing indicated A1CF △RRM mutants. (**C**). Merged image of EdU staining (red) and Hoechst staining (blue) for 786-O cells expressing indicated plasmids. (**B, D**). Statistical analysis of 786-O cell proliferation. Values were presented as mean ± SEM ($n = 3$). p-values were calculated by Student t-test, * $p < 0.05$.

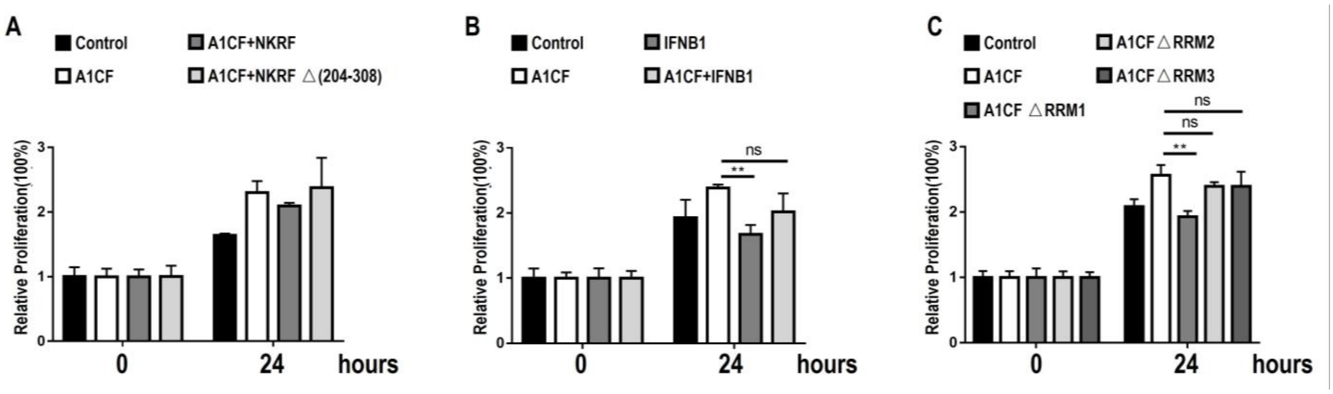

Figure S4. Disrupting the A1CF-NKRF interaction changed the renal cancer cell proliferation measured by MTT assay through A1CF-NKRF- NF-κB- IFN-β axis. (**A-C**). Proliferation of 786-O cells expressing the indicated plasmids were measured by MTT assay. Cells indicated above were seeded on 96-well plates and cell viability was measured at 0, 24 hours. Results were expressed as mean ± SEM from 6 replicate well. p-values were calculated by Student t-test, ns=no significance; $^{**}p < 0.01$.

Table S1: Primers used for plasmid constructs in this paper

Primer Name	Sequence
F-h.A1CF CDS	5'-GGATCCGAATTCATGGAATCAAATCACAAATCCG-3'
R-h.A1CF CDS	5'-ATGGTGGTGCTCGAGTCAGAAGGTGCCATATCCATC-3'
F-h.A1CF-truncation(1-293aa)	5'-GGATCCGAATTCATGGAATCAAATCACAAATCCGGGGA-3'
R-h.A1CF-truncation(1-293aa)	5'-ATGGTGATGGTGGTGTTATTAACCATCCAGCACCTTGCCAT-3'
F-h.A1CF-truncation(294-586aa)	5'-GGATCCGAATTCTCCCCCATTGAAGTCACCCTAGCAAA-3'
R-h.A1CF-truncation(294-586aa)	5'-ATGGTGATGGTGGTGTCAGAAGGTGCCATATCCATCCCCTC-3'
R-H.A1CF(1-130aa)	5'-ATGGTGATGGTGGTGTTATTAACAAACCCCTAAGAGGCGCCCATTT-3'
R-H.A1CF(131-220aa)	5'-GGATCCGAATTCGCCAGTGTGGACAACTGCCGATTAT-3'
R-H.A1CF(131-220aa)	5'-ATGGTGATGGTGGTGTTATTAACTTCTGGCTCTGCCCAGTCTCCTG-3'
F-h.A1CF(221-293aa)	5'-GGATCCGAATTCGAAGTTGATGAAGATACAATGTCT-3'
F-h.NKRF CDS	5'-GGATCCGAATTCATGGGCTTTATGTTACCTCTCATCT-3'
R-h.NKRF CDS	5'-ATGGTGATGGTGGTGTTCAATTTGCTTGAGGCATAACAAGC-3'
R-h.NKRF(1-204aa overlap)	5'-ACACACCACGAGGTCTTCTGGATTAGAAAGGTTCTTCCAG-3'
F-h.NKRF(308aa-end)	5'-GACCTCGTGGTGTGTCAGATTGGCA-3'
F-h.IFNB1 CDS	5'-GGATCCGAATTCAGGCGACACTGTTCGTGTTGTCAAC-3'
R-h.IFNB1 CDS	5'-ATGGTGATGGTGGTGCCCAGAGGCACAGGCTAGGAGATCT-3'
F-A1CF shRNA#1	5'-CCGGCCATGCTGCAAGGAGAGTATACTCGAGTATACTCTCCTTGCAGCATGGTTTTTG-3'
R-A1CF shRNA#1	5'-AATTCAAAAACCATGCTGCAAGGAGAGTATACTCGAGATACTCTCCTTGCAGCATGG-3'
F-A1CF shRNA#2	5'-CCGGGCTGCTGCTGCTACTGCTTTC CTCGAG GAAAGCAGTAGCAGCAGCAGCTTTTTG-3'
R-A1CF shRNA#2	5'-AATTCAAAAAGCTGCTGCTGCTACTGCTTTC CTCGAG GAAAGCAGTAGCAGCAGCAGC-3'

Table S2: Primers used for qPCR in this paper

Primer Name	Sequence
A1CF-s	5'-TGTCATCGTCTACCCAAGCG-3'
A1CF-as	5'-GCTCTGCCCAGTCTACTGC-3'
IFNB1-s	5'-TCTTTCCATGAGCTACAACTTGCT-3'
IFNB1-as	5'-GCAGTATTCAAGCCTCCCATTC-3'
RELA-s	5'-TTTCTCCTCAATCCGGTGAC-3'
RELA-as	5'-ACCCCTCCCTACGCAGAC-3'
NKRF-s	5'-CCAAACCTTCCAAAGGTCAA-3'
NKRF-as	5'-CAGGGTTCCCACTGTCAAAA-3'
18s-s	5'-GTAACCCGTTGAACCCCATT-3'
18s-as	5'-CCATCCAA TCGGTAGTAGCG-3'
